# Profil épidémiologique et tomodensitométrique des fractures maxillo-faciales post-traumatiques à Mopti au Mali

**DOI:** 10.11604/pamj.2022.41.309.28752

**Published:** 2022-04-18

**Authors:** Souleymane Sanogo, Alassane Kouma, Issa Cissé, Ilias Guindo, Ouncoumba Diarra, Ousmane Traoré, Mamadou Dembélé, Siaka Sidibé

**Affiliations:** 1 Service de Radiologie, Hôpital Sominé Dolo de Mopti, Sevare, Mali,; 2 Service de Radiologie, Centre Hospitalier Universitaire Mère-enfant Luxembourg, Bamako, Mali,; 3 Service de Radiologie, CHU de Kati, Kati, Mali,; 4 Service de Radiologie, CSREF CIII, Bamako, Mali,; 5 Clinique Médicale « Marie Curie », Bamako, Mali,; 6 Clinique Médicale « FERTILIA » Bamako, Mali,; 7 Service de Radiologie, Centre Hospitalier Universitaire du Point G, Bamako, Mali

**Keywords:** Fracture maxillo-faciale, lefort II, tomodensitométrie, Mopti, Maxillofacial fracture, lefort II, CT scan, Mopti

## Abstract

**Introduction:**

les traumatismes de la face constituent un problème de santé publique physiquement et psychologiquement, caractérisé par la variété des lésions et parfois par la gravité des séquelles esthétiques ou fonctionnelle. Le but de ce travail était de décrire le profil épidémiologique et tomodensitométrique des fractures maxillo-faciales à Mopti.

**Méthodes:**

étude descriptive transversale de janvier 2019 à décembre 2019 au Service de Radiologie de l´Hôpital de Mopti. Étaient inclus tous les patients ayant bénéficié une tomodensitométrie maxillo-faciale pour traumatisme avec fracture au scanner pendant cette période. Les variables analysées étaient l´âge, le sexe, l´étiologie et les types de fractures observées à la tomodensitométrie. L´enregistrement et l´analyse des données étaient faits avec les logiciels SPSS version 20 et Excel 2013.

**Résultats:**

sur un total de 120 patients, l´âge moyen était de 26,43 ans avec un écart-type de 14,547. Les hommes ont prédominé avec 75% (n= 90). Les accidents de la voie publique étaient l´étiologie fréquente avec 50% (n= 60). Les fractures occlus-faciales représentaient 38,33% (n= 46) avec une prédominance des fractures Lefort II soit 22,50% (n= 27).

**Conclusion:**

cette étude nous a permis d´identifier les couches de la population les plus touchées par les fractures maxillo-faciales dans la région de Mopti tels que les adolescents et les adultes jeunes. Le profil tomodensitométrique était dominé par les fractures occlusofaciales notamment celles de Lefort type II.

## Abstract (en)

**Introduction:**

facial injuries are a public health problem, both physically and psychologically, characterized by a variety of injuries and sometimes by severe esthetic or functional sequelae. The purpose of this study was to describe the epidemiological and tomodensitometric aspects of maxillofacial fractures in Mopti.

**Methods:**

we conducted a cross-sectional and descriptive study in the Department of Radiology of the Mopti Hospital from January 2019 to December 2019. All patients who had undergone maxillofacial CT scan for a trauma with fracture confirmed by CT scan during this period were included. The variables analyzed were age, sex, etiology and the types of fractures observed on CT scan. Data recording and analysis were carried out using SPSS version 20 and Excel 2013.

**Results:**

of a total of 120 patients, the mean age was 26.43 years with a standard deviation of 14.547. Men predominated (75%; n= 90). Road accidents were the leading cause of fractures (50%; n = 60). Occlusofacial fractures accounted for 38.33% (n= 46). Lefort II was the most common fracture (22.50%; n= 27).

**Conclusion:**

this study allowed us to identify the population groups most affected by maxillofacial fractures in the Mopti region: adolescents and young adults. Tomodensitometric results were dominated by occlusofacial fractures, in particular Lefort type II fractures.

## Introduction

Les traumatismes sont un problème mondial de santé publique constituant l´une des principales causes de morbidité et de mortalité, aussi bien dans les pays développés que dans ceux en développement [1, 2]. Parmi les nombreuses lésions traumatiques, celles de la face sont des plus fréquentes en raison de la position anatomique particulièrement exposée de cette partie du corps et de la fragilité du squelette facial [2, 3]. Les traumatismes de la face sont fréquents et concernent essentiellement la population d´adultes jeunes et de sexe masculin [4]. C´est un problème de santé publique physiquement et psychologiquement, caractérisé par la variété des lésions et parfois par la gravité des séquelles esthétiques ou fonctionnelle [5, 6]. De plus, les fractures maxillo-faciales sont souvent associées à une morbidité sévère, une perte de la fonction, une défiguration et un coût économique [7, 8]. Les accidents de la voie publique restent, avec les rixes, sa première cause [4]. La morphologie de ces fractures étant souvent complexe, la tomodensitométrie multi-détecteurs avec reformation multi-planaire (MPR) et tridimensionnelle des images est une partie standard de l'évaluation de ces blessures maxillo-faciales en raison de sa sensibilité [9]. La région de Mopti est la cinquième région administrative du Mali. Carrefour entre le Nord et le Sud du pays, les activités économiques principales sont l´agriculture, l´élevage et la pêche. Ces activités conduisent à des déplacements de population les exposant entre autres aux risques d´accident de circulation avec une recrudescence des traumatismes crâniens et maxillo-faciaux [10]. Le but de ce travail était de décrire le profil épidémiologique et tomodensitométrique des fractures maxillo-faciales dans cette région.

## Méthodes

**Conception et cadre de l'étude:** il s´agissait d´une étude descriptive transversale durant une période d´un an allant du 1^er^ janvier 2019 au 31 décembre 2019. Elle s´est déroulée dans le service d´Imagerie Médicale de l´Hôpital Sominé Dolo de Mopti. La région de Mopti est la cinquième région administrative du Mali située entre le Nord et le Sud du pays.

**Population d'étude:** la population cible a concerné tous les patients avec un traumatisme maxillo-facial ayant une fracture à la tomodensitométrie pendant la période d´étude.

**Critères d´inclusion:** tous les patients avec traumatisme facial ayant une fracture à la tomodensitométrie.

**Critères de non-inclusion:** tous les patients sans notion de traumatisme facial. Les patients avec un traumatisme et ayant un résultat tomodensitométrique normal.

**Collecte des données:** les données sociodémographiques et les renseignements indispensables pour cette étude étaient recueillis à travers des formulaires préétablis à cet effet. Cette récollette de données était faite à partir du bulletin de demande de l´examen tomodensitométrique de chaque patient ou par l´interrogatoire direct du patient ou de ses parents à la quête de complément d´information. Les résultats tomodensitométriques étaient directement portés sur les formulaires après l´analyse des images sur la console.

**Matériel et protocole d´examen:** l´examen tomodensitométrique a été réalisé sur un appareil de 16 barrettes, Somatom Emotion de marque Siemens. L´exploration a été réalisée sans injection intraveineuse de produit de contraste. Les images ont été analysées en fenêtre parenchymateuse et osseuse.

**Variables étudiées:** l´âge, le sexe, les mécanismes étiologiques du traumatisme et les différents types de fractures maxillo-faciales observées à la tomodensitométrie.

**Analyse statistique:** l´enregistrement et l´analyse des données ont été faits avec les logiciels SPSS version 20 et Excel 2013. Nous avons calculé des fréquences et des pourcentages. Les statistiques de tendance centrale et de dispersion calculées étaient la moyenne et l´écart type de façon respective.

**Considérations éthiques:** les participants à l'étude ont donné leurs consentements éclairés. L´anonymat des patients pendant la collecte des données était une obligation. La confidentialité des résultats de chaque patient était respectée.

## Résultats

### Profil sociodémographique

Au total, 120 patients ont été colligés. La tranche d´âge la plus fréquente était de 15 à 29 ans avec 30,83% (n= 37) suivie de celle de 30 à 44 ans avec 25% (n= 30). L´âge moyen était de 26,43 ans avec un écart-type de 14,479. Les hommes ont prédominé notre série avec 75% (n= 90) avec un sexe ratio de 3 (Tableau 1).

**Tableau 1:** données sociodémographiques

Données sociodémographiques	n	%
**Tranches d´âge en année**		
≤14	8	6,67
15-29	37	30,83
30-44	30	25,00
45-59	24	20,00
60-74	15	12,50
75 ≥	6	5,00
Sexe		
Masculin	90	75,00
Féminin	30	25,00

**Mécanismes étiologiques:** les accidents de la voie publique étaient le mécanisme étiologique le plus fréquent avec 50% (n= 60). Les coups et blessures volontaires étaient au deuxième rang avec 22,50% (n= 27) (Tableau 2).

**Tableau 2:** répartition des patients en fonction du mécanisme étiologique

Mécanisme étiologique	n	%
Accident de la voie publique	60	50,00
Coups et blessures volontaires	27	22,50
Chute de hauteur	6	5,00
Conflit armé	23	19,17
Autres	4	3,33
Total	120	100

**Fractures observées à la tomodensitométrie:** les fractures occlusofaciales étaient plus fréquentes soit 38,33% (n= 46) avec une prédominance des fractures de Lefort II soit 22,50% (n= 27). Les fractures latéro-faciales occupaient le deuxième rang avec 27,5% (n= 33) notamment au niveau de l´arc zygomatique soit 12,50% (n= 15) (Tableau 3).

**Tableau 3:** types de fractures maxillo-faciales à la tomodensitométrie

Types de fractures	n	%
Fracas maxillo-facial	11	9,17
Fractures mandibulaires isolées	18	15,00
Fractures du bandeau frontal	4	3,33
**Fractures latéro-faciales**		
Fractures du trépied zygomatique	10	8,33
Fractures de l´arc zygomatique	15	12,50
Fractures du corps zygomatique	2	1,67
Fractures de l´arcade frontozygomatique	6	5,00
**Fractures occlusofaciales**		
Lefort I	9	7,50
Lefort II	27	22,50
Lefort III	6	5,00
Disjonction intermaxillaire	4	3,33
**Fractures centro-faciales**		
Fractures des os propres du nez	6	5,00
Fracture du CNEMFO (Complexe Naso-Ethmoïdo-Maxillo-Fronto-Orbitaire)	2	1,67
Total	120	100

**Figure 1:**
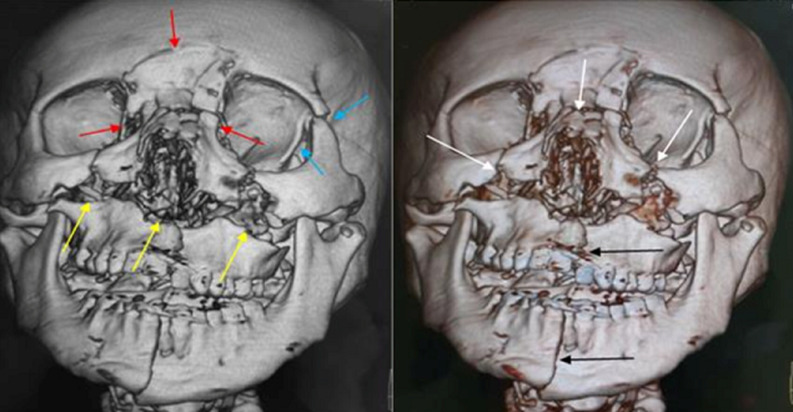
images tomodensitométriques maxillo-faciales, reconstructions 3D en vue de face

**Figure 2:**
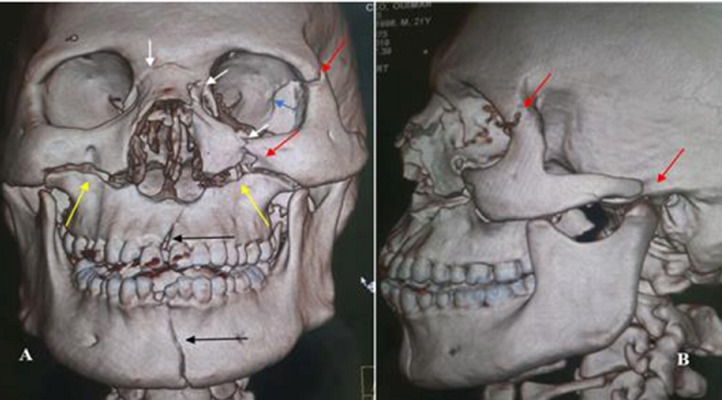
images tomodensitométriques maxillo-faciales, reconstructions 3D en vue de face (A) et latérale gauche (B)

**Figure 3:**
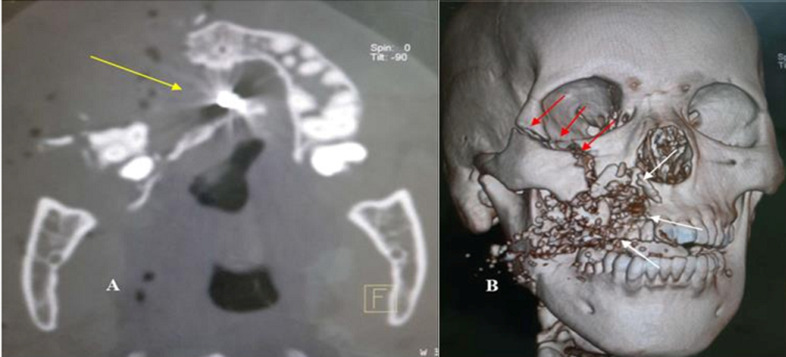
images tomodensitométriques maxillo-faciales, coupe axiale en fenêtre osseuse (A) et reconstruction 3D (B)

**Illustrations iconographiques:** les Figure 1, Figure 2, Figure 3 sont des illustrations iconographiques tomodensitométriques en reconstructions 3D et en coupe axiale fenêtre osseuse objectivant des fractures maxillo-faciales.

## Discussion

Le but de ce travail était de décrire le profil épidémiologique et tomodensitométrique des fractures maxillo-faciales à Mopti. L´épidémiologie des traumatismes maxillo-faciaux varie selon les caractéristiques démographiques, les habitudes culturelles, l´environnement industriel, les moyens de transport, le climat politique, la législation du pays [7, 11]. Les fractures maxillo-faciales est un terme qui englobe de nombreux différents types de fractures impliquant les os du visage [12].

La tranche d´âge la plus fréquente était de 15 à 29 ans avec 30,83%. Les hommes ont prédominé notre série avec 75% soit un sexe ratio de 3. Les traumatismes maxillo-faciaux touchent principalement l´homme jeune, soit une moyenne de 30 ans [7, 11]. Selon une étude japonaise, la tranche d´âge la plus touchée par les traumatismes maxillo-faciaux se situe entre 10 et 20 ans [13]. Au Cameroun, Messina *et al*. rapportent que le sexe masculin représentait 91% [14]. La tranche d´âge la plus représentée était celle de 20-29 ans avec un âge moyen de 25,1 ans [14]. Au Mali, Sangaré M rapporte dans son travail que la tranche d´âge la plus représentée était celle comprise entre 16 et 30 ans avec une moyenne d´âge de 22,30 ans [15]. Le sexe masculin a dominé avec 78,90% avec un sex-ratio de 3,75 [15]. Selon Samaké S, une prédominance des patients de 16-30 ans était notée dans 44,83% avec une d´âge à 27,45 ans et le sexe masculin représentait 85,30% [6].

Dans notre étude, les accidents de la voie publique étaient le mécanisme étiologique le plus fréquent avec 50% suivis des coups et blessures volontaires (CBV) soit 22,50%. Dans son étude, Sangaré M rapporte que les accidents de la voie publique ont prédominé avec 80,3% [15]. Sasaki *et al*. ont indiqué que 31% des fractures maxillo-faciales étaient dues à des accidents de la route, 29% à des chutes accidentelles, 23% à la violence et 14% au sport [16]. Velayutham *et al*. ont montré que la cause la plus fréquente des blessures maxillo-faciales chez les personnes âgées était les chutes, tandis que chez les plus jeunes, il s'agissait de violence interpersonnelle [17]. Les fractures maxillo-faciales chez les personnes âgées sont moins fréquentes que chez les plus jeunes [18]. Dans notre contexte, les moyens de déplacement privilégiés sont les engins à quatre roues et à deux roues notamment les motocyclettes et les charrettes. Ceux-ci exposent la population entre autres aux risques des accidents routiers et aux risques sécuritaires. Les tensions entre certaines communautés et l´accroissement du banditisme sont des facteurs exposant cette population aux risques des CBV.

L'imagerie, notamment la tomodensitométrie, permet d'identifier les fractures non décelées à l'examen physique. Elle permet de mieux décrire et de classifier les fractures. Ceci aide dans la prise de décision thérapeutique [12]. Dans notre étude, les fractures occlusofaciales étaient plus fréquentes (38,33%) avec une prédominance des fractures de Lefort II soit 22,50%. Les fractures latéro-faciales occupaient le deuxième rang (27,5%) notamment au niveau de l´arc zygomatique, soit 12,50%. Samaké rapporte une fréquence élevée des fractures latéro-faciales avec 49,1% avec une prédominance des fractures maxillaires (47,77%) des fractures latéro-faciales. Ce même auteur avait observé une prédominance des fractures de Lefort I et II avec 41,37% chacune parmi les fractures transversales [6]. L´os zygomatique était le site anatomique le plus fréquent chez les hommes et les femmes avec 34%, suivi de la mandibule avec 25% [19]. Sangaré avait trouvé une prédominance des fractures mandibulaires avec 38,2% [15]. Chez Sasaki *et al*. les fractures mandibulaires étaient au premier plan avec 87%, suivies des fractures maxillaires (14%) et les fractures zygomatiques avec 12% [16]. Bouguila *et al*. avait trouvé également une prédominance des fractures mandibulaires avec 62% [11]. La topographie des fractures maxillo-faciales à la tomodensitométrie varie d´un auteur à l´autre dans la littérature révisée. Ceci pourrait être en fonction de la taille de l´échantillon de chaque auteur et des réalités sociodémographiques de chaque région géographique.

Notre étude a pour limite d´être intra-hospitalière prenant uniquement en compte les patients avec un traumatisme maxillo-facial et présentant une fracture à l´exploration tomodensitométrique pendant la période d´étude. Cependant, cette étude est une première dans le domaine de l´imagerie médicale à l´Hôpital de Mopti. Elle constitue désormais une référence pour les études ultérieures.

## Conclusion

Cette étude nous a permis d´identifier les couches de la population les plus touchées par les fractures maxillo-faciales dans la région de Mopti tels que les adolescents et les adultes jeunes. Le profil tomodensitométrique était dominé par les fractures occlusofaciales notamment celles de Lefort type II. La tomodensitométrie demeure une pierre angulaire dans le diagnostic des fractures maxillo-faciales.

### Etat des connaissances sur le sujet

- Les lésions traumatiques de la face sont fréquentes en raison de sa position anatomique;
- C´est un problème de santé publique physiquement et psychologiquement;
- La tomodensitométrie est incontournable dans le diagnostic des fractures maxillo-faciales.

### Contribution de notre étude à la connaissance

- C´est une première étude dans le domaine de l´imagerie médicale à Mopti;
- Elle constitue désormais une référence pour les études ultérieures;
- Les fractures maxillo-faciales sont plus fréquentes chez les adolescents et les adultes jeunes.

## Remerciements

Nos remerciements vont à l´endroit de Mr Ibrahim Badian Konaré, Mr Soungalo Dembélé, Mr Paul Diarra, Mr Hamidou Sidibé et Mr Adama Traoré, tous assistants médicaux en imagerie médicale en service à l´Hôpital Sominé Dolo de Mopti pour leur soutien à l´élaboration de ce travail.
